# Enhanced inhibition of clonogenic survival of human medulloblastoma cells by multimodal treatment with ionizing irradiation, epigenetic modifiers, and differentiation-inducing drugs

**DOI:** 10.1186/s13046-016-0376-1

**Published:** 2016-06-17

**Authors:** Ina Patties, Rolf-Dieter Kortmann, Franziska Menzel, Annegret Glasow

**Affiliations:** Department of Radiation Therapy, University of Leipzig, Stephanstraße 9a, Leipzig, 04103 Germany; Institute of Anatomy, University of Leipzig, Liebigstraße 13, 04103 Leipzig, Germany

## Abstract

**Background:**

Medulloblastoma (MB) is the most common pediatric brain tumor. Current treatment regimes consisting of primary surgery followed by radio- and chemotherapy, achieve 5-year overall survival rates of only about 60 %. Therapy-induced endocrine and neurocognitive deficits are common late adverse effects. Thus, improved antitumor strategies are urgently needed. In this study, we combined irradiation (IR) together with epigenetic modifiers and differentiation inducers in a multimodal approach to enhance the efficiency of tumor therapy in MB and also assessed possible late adverse effects on neurogenesis.

**Methods:**

In three human MB cell lines (DAOY, MEB-Med8a, D283-Med) short-time survival (trypan blue exclusion assay), apoptosis, autophagy, cell cycle distribution, formation of gH2AX foci, and long-term reproductive survival (clonogenic assay) were analyzed after treatment with 5-aza-2′-deoxycytidine (5-azadC), valproic acid (VPA), suberanilohydroxamic acid (SAHA), abacavir (ABC), all-*trans* retinoic acid (ATRA) and resveratrol (RES) alone or combined with 5-aza-dC and/or IR. Effects of combinatorial treatments on neurogenesis were evaluated in cultured murine hippocampal slices from transgenic nestin-CFPnuc C57BL/J6 mice. Life imaging of nestin-positive neural stem cells was conducted at distinct time points for up to 28 days after treatment start.

**Results:**

All tested drugs showed a radiosynergistic action on overall clonogenic survival at least in two-outof-three MB cell lines. This effect was pronounced in multimodal treatments combining IR, 5-aza-dC and a second drug. Hereby, ABC and RES induced the strongest reduction of clongenic survival in all three MB cell lines and led to the induction of apoptosis (RES, ABC) and/or autophagy (ABC). Additionally, 5-aza-dC, RES, and ABC increased the S phase cell fraction and induced the formation of gH2AX foci at least in oneout-of-three cell lines. Thereby, the multimodal treatment with 5-aza-dC, IR, and RES or ABC did not change the number of normal neural progenitor cells in murine slice cultures.

**Conclusion:**

In conclusion, the radiosensitizing capacities of epigenetic and differentiation-inducing drugs presented here suggest that their adjuvant administration might improve MB therapy. Thereby, the combination of 5-aza-dC/IR with ABC and RES seemed to be the most promising to enhance tumor control without affecting the normal neural precursor cells.

## Background

Medulloblastoma (MB) is the most common malignant brain tumor (WHO °IV) in children aged < 15 years [1]. The MB standard therapy consists of primary tumor surgery followed by radiation therapy and/or chemotherapy. Current therapy regimes are mainly guided by tumor histology, metastasic disease, extend of resection, and patient age. New trials additionally include molecular factors like ß-catenin protein and *MYC/MYCN* gene amplification status. Patients with metastases receive a more intensive treatment compared to metastases-free patients. No radiation therapy but a more aggressive chemotherapy is given to children below 4 years to avoid radiation-related adverse late effects, like neuroendocrine and neurocognitive deficits. The 5-year overall survival of approximately 60 % implies the urgent need of improved antitumor therapies to enhance the outcome especially of high-risk patients (infants, metastatic disease: ~ 55 % of all MB patients).

During embryonal development, MB arises from neural precursor cells in different zones of the rhombic lip, wherefrom they are growing into the cerebellum or the brain stem [2]. It is widely believed that tumor formation is initiated by genetic, gene-regulatory, or epigenetic abnormalities, which inhibit the normal neuronal or glial differentiation [3]. In 70–90 % of primary MBs, hypermethylation of gene promotors of tumor suppressor genes (TSG) is observed, which leads to their inactivation and, finally, to unrestricted proliferation and blockage of apopotosis [3]. Hence, the application of approved epigenetic modifiers, like 5-aza-2′-deoxycytidine (5-aza-dC, decitabine), valproic acid (VPA), or suberanilohydroxamic acid (SAHA, Vorinostat®), which have been shown by us [4] and others [5–8] to demethylate TSG, seems to be a suitable approach to inhibit tumor cell growth. These substances induce a cell cycle arrest at G2/M [9–11], where cells are most radiosensitive (reviewed in [12]). Therefore, synergistic effects with IR might be anticipated. Besides, MBs are mostly poorly differentiated tumors [13, 14] containing 6–21 % potential tumor stem cells (TSC) [15], which are often chemo- and radiotherapy-resistant (reviewed in [16]) and held responsible for tumor relapse (reviewed in [17]). Differentiation-inducing drugs like all-*trans* retinoic acid (ATRA), abacavir (ABC), or resveratrol (RES) are applied in this study for their potential to induce the maturation of MB tumor stem cells and, thereby, to suppress their cancer-forming capacities (previously described in [18]). Besides, ATRA is able to inhibit MB cell growth by suppression of the *OTX2* (*orthodenticle homeobox 2*) gene, which is overexpressed in the majority of medulloblastomas [19]. In addition, tumor cell-specific activity might be exerted by ABC through telomerase inhibition, which is enhanced in 70 % of MBs [20] and by RES through its Cu(II)-dependent oxidative effects, which may induce cell death especially in low pH environments. As tumor cells are often acidic due to their glycolysis dependence and have elevated copper levels, they might be more sensitive to RES than normal cells [21].

Recently, we tested single and combinatorial effects of the *de novo* methyltransferase (DNMT) inhibitor 5-aza-dC with IR [4] or with other epigenetic/differentiation-inducing drugs on the metabolic activity and reproductive survival of human MB cells [18]. Here, we combined for the first time IR, an integral part of MB standard therapy in children > 4 years, with 5-aza-dC and previously evaluated [18] drugs (VPA, SAHA, RES, ABC, ATRA) in a multimodal approach to further enhance the efficiency of MB tumor therapy. As brain tumor treatment is known to induce cognitive deficits by inhibition of hippocampal neurogenesis [22], also potential adverse effects have been evaluated using a murine entorhino-hippocampal slice culture model.

## Methods

All methods were performed at room temperature unless otherwise noted.

### Modulators

5-Aza-dC (trade name Dacogen®), ATRA, RES, and VPA were purchased from Sigma-Aldrich (Munich, Germany). ABC hemisulfate was kindly provided from GlaxoSmithKline (Hamburg, Germany) and SAHA (trade name Zolinza®) from MSD (Haar, Germany). Stock solutions were prepared as follows and stored at - 20 °C: 10 mM 5-aza-dC in PBS; 500 μM ATRA in 10 % ethanol (stored at - 80 °C); 500 μM RES in 1 % ethanol; 1 M VPA in PBS; 100 mM ABC in PBS; 100 μM SAHA in 0.25 % DMSO. Further working solutions were made in PBS and equal volumes were administered into the culture medium. To exclude effects based on ethanol or DMSO applications, appropriate controls were implemented. Drug concentrations of 5-aza-dC, SAHA, ATRA, and RES applied here are guided by achievable serum levels [23–26]. However, concentrations of VPA and ABC are 2- to 25-fold higher than tested in pharmacokinetic studies until now [27, 28].

### Cell lines

The human MB cell line MEB-Med8a was kindly provided by Prof. T. Pietsch (Department of Neuropathology, University of Bonn Medical Centre, Bonn, Germany). The MB cell lines D283-Med and DAOY were purchased from ATCC cell biology collection (Manassas VA, USA). DAOY cells are *TP53*-mutated, whereas MEB-Med8a and D283-Med cell lines are wild-type *TP53* [29, 30]. D283-Med and DAOY were maintained in MEM (Sigma-Aldrich, Munich, Germany) including 2 mM L-glutamine (Biochrom, Berlin, Germany), MEB-Med8a in DMEM with 4.5 g glucose (Lonza, Basel, Switzerland), all supplemented with 10 % fetal calf serum (PAA, Yeovil, Somerset, UK), 100 U/ml penicillin, and 100 μg/ml streptomycin (Biochrom, Berlin, Germany) at 37 °C and 5 % CO_2_ unless otherwise noted.

### Animals

Mouse breeding was performed in the animal facility of the Faculty of Medicine, University of Leipzig according to European (Council Directive 86/609/EEC) and German (Tierschutzgesetz) guidelines for the welfare of experimental animals. Nestin-CFPnuc C57BL/J6 mice, generated by Encinas et al. [31] were housed in a 12 h/12 h light/dark cycle with access to food and water *ad libitum*.

### Preparation of murine entorhino-hippocampal slice cultures

Organotypic entorhino-hippocampal slice cultures were generated from nestin-CFPnuc C57BL/J6 mice, postnatal day (p) 3 to 6 as initially described by Gahwiler et al. [32] and modified by Kluge et al. [33]. Briefly, after decapitation of the mice, brains were removed and 350 μm-thick horizontal slices were prepared on a vibratome (Leica VT 1000). The entorhino-hippocampal structure was then resected and transferred onto membrane inserts (Millicell PICMORG50, Millipore) in six-well cell culture plates. Tissue slices were cultivated at 37 °C and 5 % CO_2_ and cell culture medium (MEM, Invitrogen; 25 % HBSS, Invitrogen; 25 % horse serum, Invitrogen; 1 % L-glutamine, Sigma; 1 % penicillin/streptomycin, Lonza; 1 % glucose, Applichem) was renewed three times a week. Slices were cultivated for 7-14 days before start of the experiment to allow tissue healing.

### Single drug and multimodal treatment of cell lines

To examine concentration effects of the single drugs, cells were seeded in cell culture flasks and five different concentrations of the modulator were added after 24 h and again at medium renewal 4 days after seeding. For combination experiments, cells were seeded and, 24 h later, treated simultaneously with IC 90 of 5-aza-dC and a second drug (concentrations see Table 1). After three treatment days, cells were irradiated with 2 or 8 Gy, medium was renewed, and 5-aza-dC and the second drug were supplemented. After overall six treatment days, cells were seeded for clonogenic assay.

**Table 1:** Drug concentrations for the combinatorial treatment of human MB cells

cell line	D283-Med	MEB-Med8a	DAOY
5-aza-dC	0.05 μM	0.1 μM	0.5 μM
VPA	3 mM	1 mM	2 mM
SAHA	1 μM	2 μM	2 μM
Abacavir	0.25 mM	0.04 mM	0.24 mM
ATRA	1.5 μM	10 μM	10 μM
Resveratrol	11 μM	9.5 μM	40 μM

For combination experiments, IC 90 values were calculated from single drug clonogenic assays for each cell line separately and indicate the drug concentration leading to 90 % inhibition of colony formation. For SAHA and ATRA, the maximal tolerated plasma concentrations were used in MEB-Med8a and DAOY, leading to IC 71/IC 82 (SAHA) or IC 46/IC 72 (ATRA), respectively

### Single drug and multimodal treatment of tissue slices

To assess the neurotoxic potential of the most promising multimodal settings, murine enthorino-hippocampal tissue slices were treated (day 0) with 0.5 μM 5-aza-dC, 40 μM RES or 250 μM ABC, corresponding to the maximal IC 90 in the three cell lines. After 72 h, single dose irradiation with 8 Gy was performed, medium was renewed, and the drugs were supplemented for additional 72 h as done for the clonogenic assay. Ethanol control contains 0.023 % ethanol which corresponds to the ethanol concentration in the resveratrol treated samples.

### Irradiation

Cell/slice cultures were irradiated with a single dose using a 150 kV X-ray machine (DARPAC 150-MC) at a dose rate of 0.86 Gy/min.

### Relative number of vital cells

To determine the number of vital cells, cells were counted using the trypan blue exclusion assay. The *relative number of vital cells* is calculated from vital cell counts after treatment normalized to the initially seeded vital cell number.

### Cell cycle distribution

To examine changes on cell cycle distribution 24 h and 72 h after treatment, cells were harvested, washed twice with PBS, and fixed with 70 % ethanol at 4 °C overnight. Afterwards, cells were washed twice in PBS, incubated with RNAseA solution (0.1 mg/ml) 20 min at 37 °C, and stained with propidium iodide (50 μg/ml) 5 min at 4 °C. The DNA staining by propidium iodide and the cell cycle distribution were then assessed by flow cytometry (Beckman coulter, EPICS XL) using EXPO32 software.

### DNA double-strand break detection

To detect DNA double-strand breaks, nuclear staining of gH2AX repair protein was executed after 1 h/24 h treatment or 1 h/24 h after IR. Cells were seeded on chamber slides (DAOY, MEB-Med8a; adherent cells) or in 6-well cell culture plates (D283-Med, suspension cells) and irradiated (8 Gy) or treated with IC 90 of 5-aza-dC, RES, or ABC (concentrations see Table 1) 24 h later. For staining, D283-Med cells were harvested by scratching and transferred to 96-well U bottom plates. Cells were fixed with 2 % formaldehyde in PBS for 15 min washed three times with PBS and permeabilized with 0.2 % Triton-X in PBS containing 1 % bovine serum albumine (PBS-BSA) for 5 min on ice. Then, cells were washed once with PBS-BSA, blocked with 3 % BSA in PBS for 1 h, and incubated with 10 μg/ml mouse anti-phospho histone H2A.X (Ser 139) antibody (clone JBW301; Millipore) or corresponding mouse IgG 1 isotype control (Acris), diluted in PBS-BSA containing 0.5 % Tween 20 (PBS-BSA-Tween), for 1 h. After three washes with PBS-BSA-Tween, cells were incubated with 2 μg/ml secondary antibody (goat anti-mouse Alexa 568 IgG F(ab’)2; Invitrogen), diluted in PBS-BSA-Tween for 1 h in the dark. Then, cells were washed three times with PBS-BSA-Tween, counterstained with DAPI (0.5 μg/ml) 5 min, cytospins prepared for D283-Med cells and mounted (fluorescence mounting medium, DAKO).

DNA double-strand breaks (DSB) were analyzed in 50 cells for each treatment. A maximum of 30 gH2AX-foci per nucleus were counted by fluorescence microscopy.

### Apoptosis

Apoptotic cells were determined using the FAM-FLICA™ in vitro Caspase 3/7 Detection Kit (Immunochemistry Technologies) according to the manufacturers’ instructions. In brief, 18 h after IR with 2 Gy or treatment with IC 90 of 5-aza-dC, RES, or ABC (concentrations see Table 1), cells were harvested and stained with FLICA™ solution for 60 min at 37 °C. After washing three times, cells were counterstained with propidium iodide (2 μg/ml) and analyzed by flow cytometry (Beckman coulter, EPICS XL).

In addition, the PI staining of sub-G1 cells (see *cell cycle distribution* in method section) was quantified as a second detection method for apoptotic cells.

### Autophagy

Autophagy was analyzed with the Cyto-ID® Autophagy Detection Kit (ENZO), according to the manufacturers’ instructions. The CytoID® detection reagent, a cationic amphiphilic tracer (CAT) selectively labels pre-autophagosomes, autophagosomes and auto(phago)lysosomes with negligible staining of lysosomes. Rapamycin and chloroquine were applied as positive and negative controls. Cells were harvested 18 h after irradiation (2 Gy) or treatment with IC 90 of 5-aza-dC, RES, or ABC (concentrations see Table 1) and stained with Cyto-ID® solution for 30 min at 37 °C before flow cytometric analysis.

The autophagy activity factor (AAF) was calculated as follows:

$$ \mathrm{A}\mathrm{A}\mathrm{F} = 100 \times \frac{{\mathrm{MFI}}_{\mathrm{treated}}\hbox{-}\ {\mathrm{MFI}}_{\mathrm{control}}}{{\mathrm{MFI}}_{\mathrm{control}}} $$

AAF autophagy activity factor

MFI mean fluorescence intensity

### Clonogenic survival

Subsequently to cell treatment, vital cells were counted using trypan blue exclusion test and seeded at two different cell densities in triplicates in 6 well cell culture plates. Normal medium without modulators was added. Ten to 14 days later, colonies were washed with PBS, fixed with ice-cold ethanol/acetone (1:2) for 10 min, stained with Giemsa solution (1:2 with distilled water) for 5 min, and washed with distilled water. Colonies with > 50 cells were counted; the quotient of counted colonies to seeded cells determined the plating efficiency (PE). The ratio between PE of treated cells and PE of untreated cells represented the clonogenic surviving fraction (SF) of seeded cells. The *overall clonogenic survival* was calculated from the relative number of vital cells multiplied with the SF.

### ‘Life imaging’ analysis of neural progenitor cells

Nestin-positive neural stem cells [34] were visualized by their nuclear-expressed cyan fluorescent protein (CFPnuc) using a confocal ‘life imaging’ microscope (Olympus IX81). Slices were imaged at 20-fold magnification at day 0 (before treatment), day 7, day 14, day 21 and 28 after treatment start. Z-stacks of three images at 4 μm intervals were prepared and fluorescent cells were quantified by ImageJ (https://imagej.nih.gov/ij/).

### Statistics

Statistical analysis of two treatment groups within a single cell line or in tissue slice experiments was conducted with parametric two-tailed, paired Student’s *t*-test using Microsoft Excel 2003 software. The number of experiments/number of animals executed is given as “n”. Joint analysis of the three cell lines was conducted with the non-parametric Wilcoxon rank sum test using SigmaPlot 11.0. In the joined analysis “n” represents the number of cell lines. Values are presented as mean ± SEM, if not otherwise noticed. *P*-values ≤ 0.05 (*; ^#^) and ≤ 0.01 (**; ^##^) were considered as statistically significant; *p*-values ≤ 0.001 (***; ^###^) as highly statistically significant.

Drug interaction analyses regarding synergistic or additive effects were conducted as described previously [18] using the Bliss independence (BI) model which is based on the non-interaction theory [35, 36].

## Results

### Short- and long-term survival of tumor cells

**Fig. 1:**
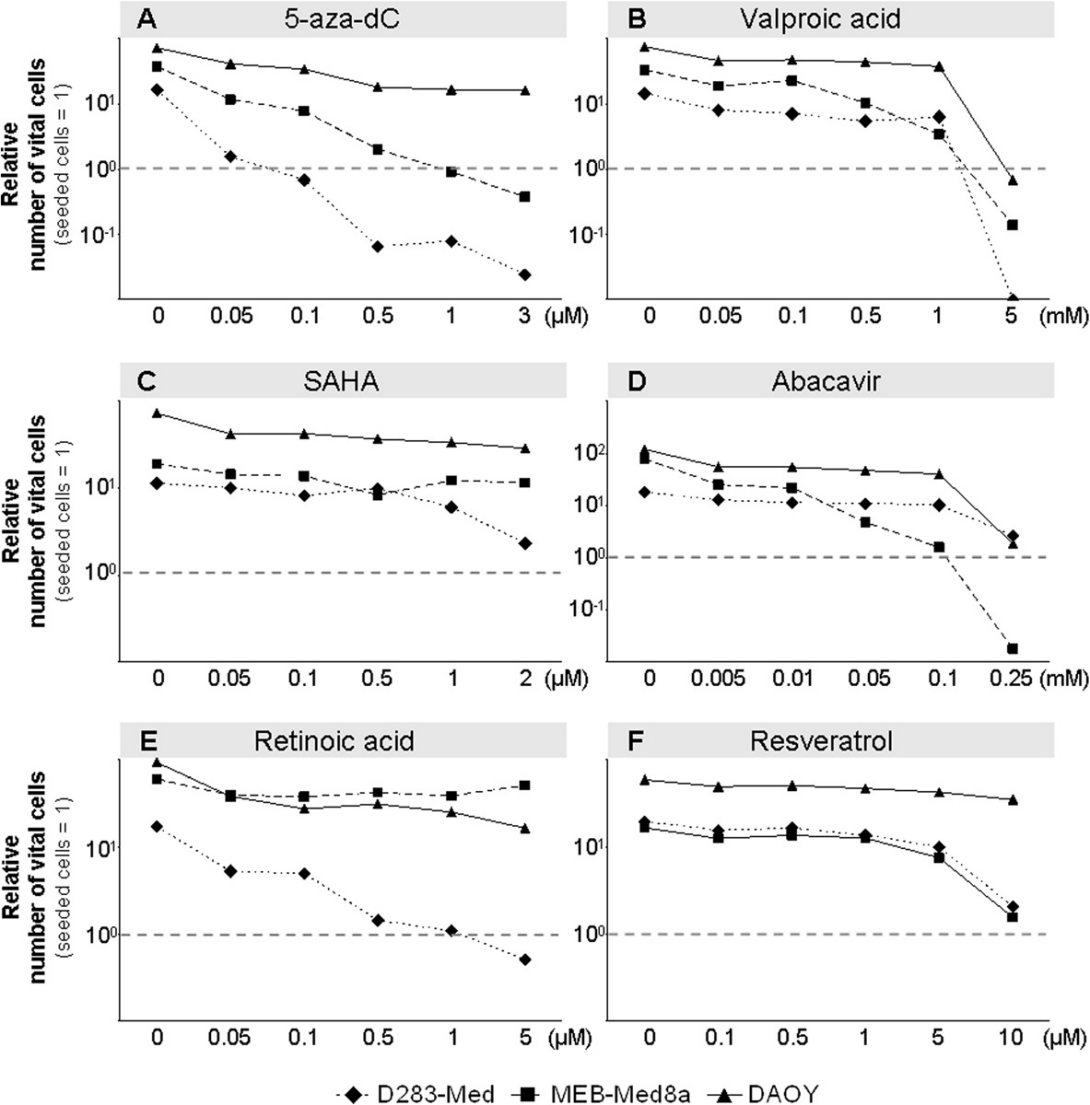
Relative number of vital MB cells after 6-day single drug treatment. The number of vital cells was examined by trypan blue exclusion assay in three MB cell lines after 6-day treatment with: **a**) 5-aza-dC, **b**) VPA, **c**) SAHA, **d**) ATRA, **e**) ABC, **f**) RES. Five distinct drug concentrations were applied and values are normalized to the initial-seeded cell number (dashed line). For each cell line one experiment was performed

**Fig. 2:**
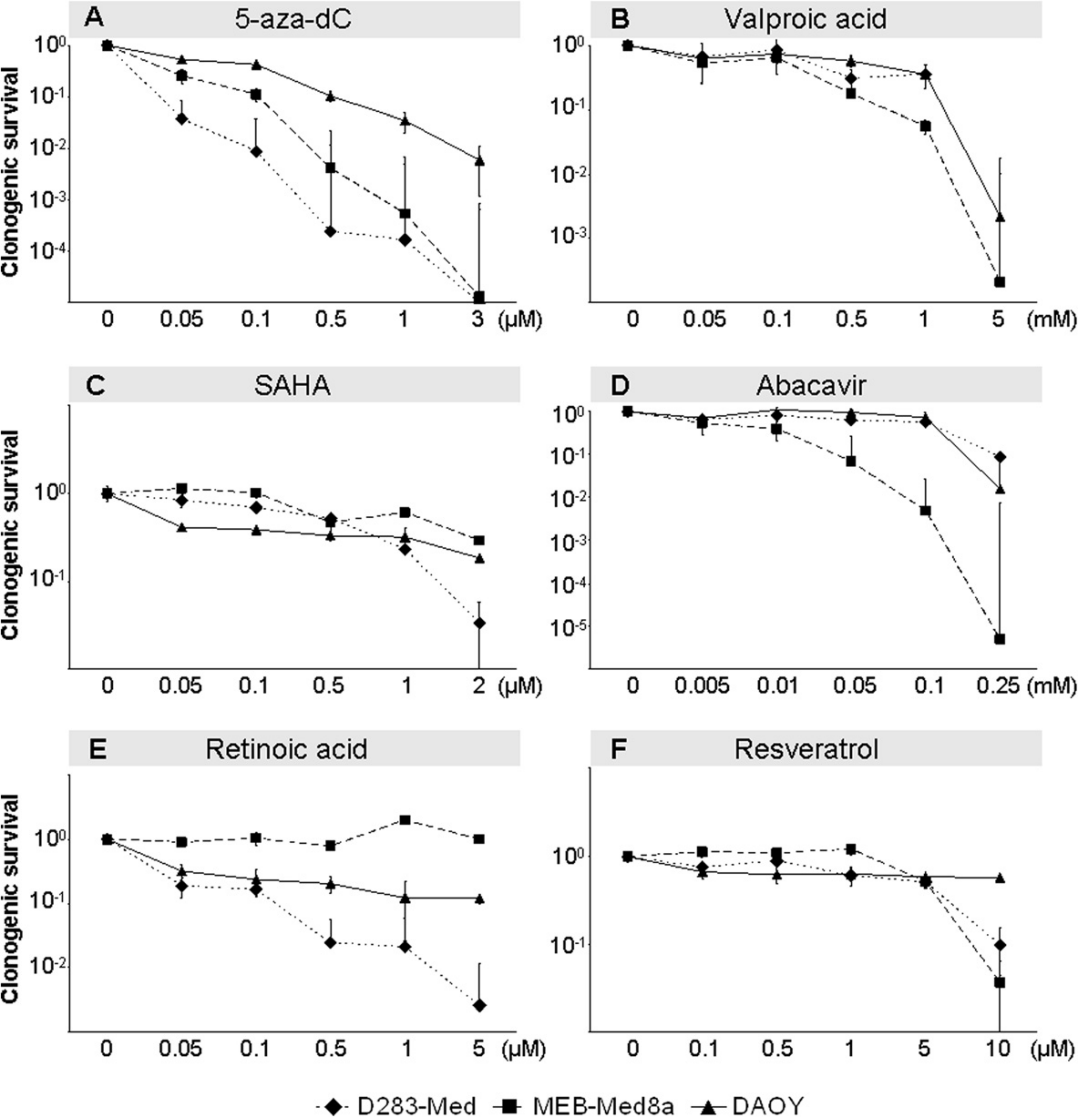
Clonogenic survival of MB cells after 6-day single drug treatment. Reproductive long-term survival was determined by clonogenic assays in three MB cell lines after 6-day treatment with: **a**) 5-aza-dC, **b**) VPA, **c**) SAHA, **d**) ATRA, **e**) ABC, **f**) RES. Five distinct drug concentrations were applied and values are normalized to untreated control. The experiment was performed once in sextuplicates. Data are presented as mean ± SEM. IC 90 values for combination experiments were calculated using Excel software and are shown in Table 1

**Fig. 3:**
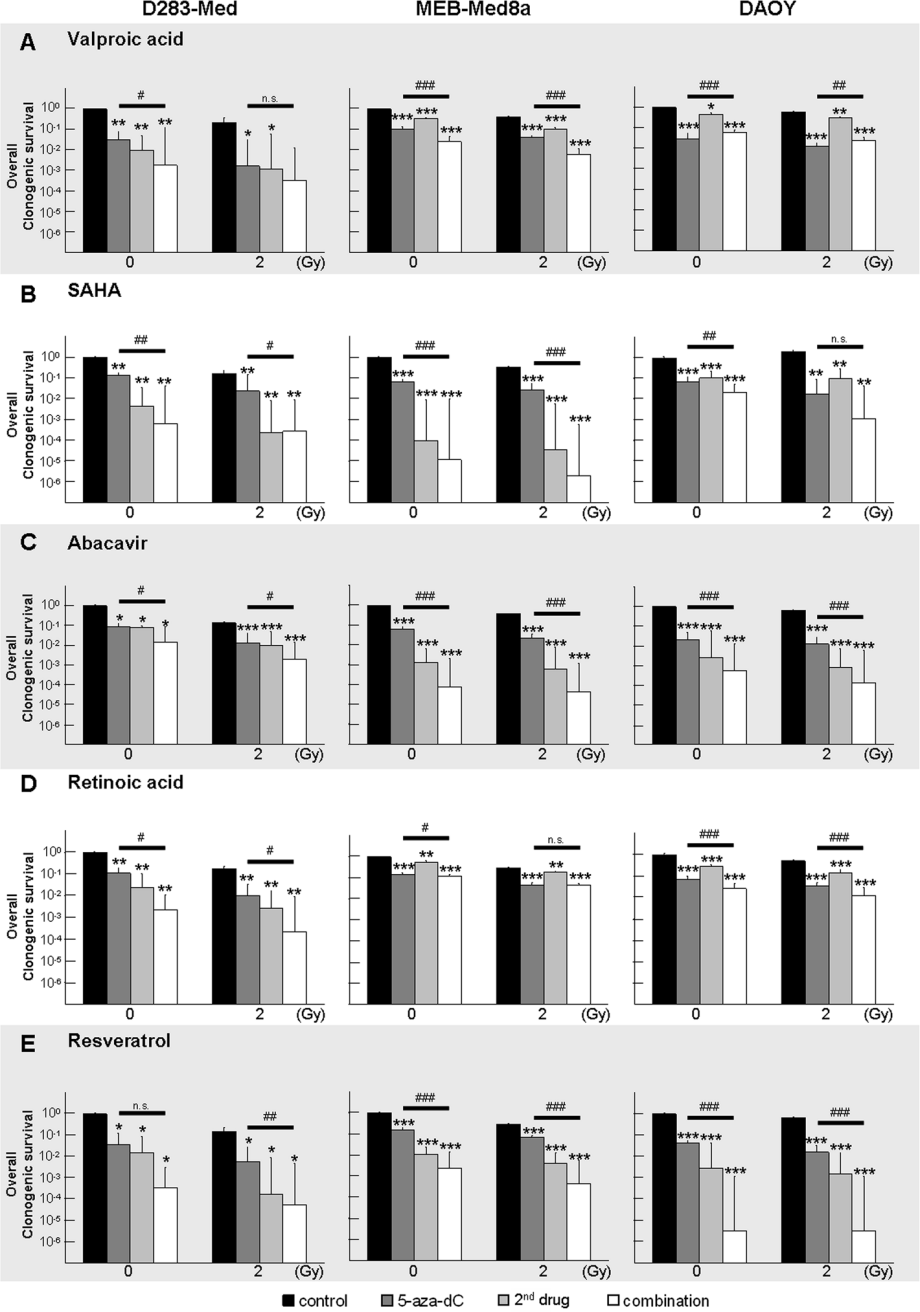
Individual presentation of overall clonogenic survival in D283-Med, MEB-Med8a and DAOY after combined treatment with 5-aza-dC, IR with 2 Gy, and **a**) VPA, **b**) SAHA, **c**) ATRA, **d**) ABC, **e**) RES. Cells were treated with 5-aza-dC and a second drug (concentrations are shown in Table 1) for 3 days pre- and 3 days post-IR (2 Gy). Clonogenic survival was examined 10–14 days later. Results after 8 Gy irradiation were similar (Table 2). Data were normalized to the untreated, nonirradiated control (=1) and relative values presented as mean ± SEM from sextuplicates. Statistical significances of treatment groups *vs.* control (0 or 2 Gy) are indicated by asterisks (*, *p* ≤ 0.05; **, *p* ≤ 0.01; ***, *p* ≤ 0.001) and for combination of all treatments (white bar) *vs.* 5-aza-dC + control by hash (#, respectively). Additional information about significant synergism/additivity and intergroup significances is given in Table 2

**Fig. 4:**
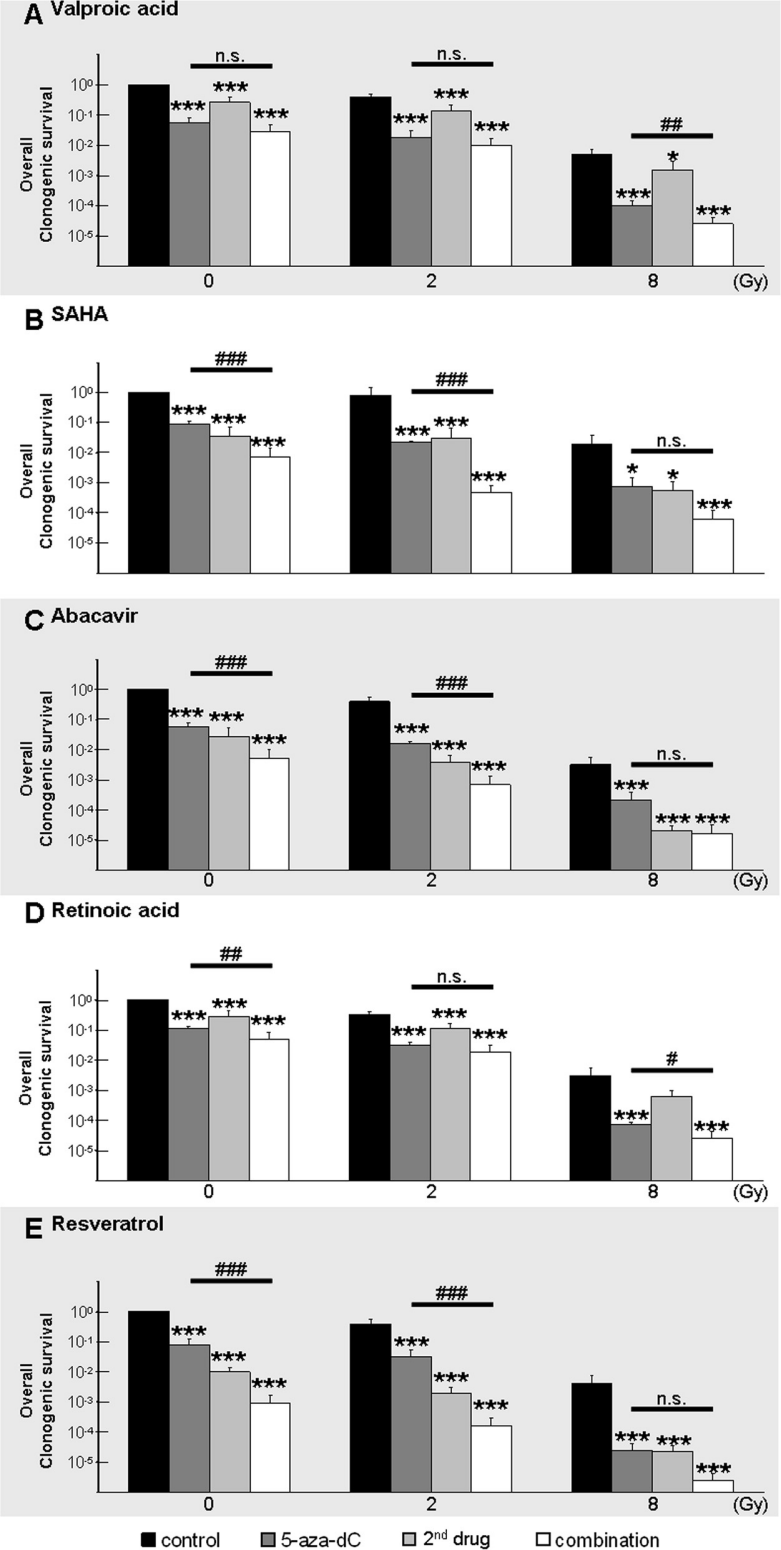
Joint analysis of clonogenic survival in all three cell lines after combined treatment with 5-aza-dC, IR, and **a**) VPA, **b**) SAHA, **c**) ATRA, **d**) ABC, **e**) RES . Relative data (untreated, nonirradiated control = 1) are presented as mean ± SEM, *n* = 3. Statistical significance is presented *vs.* control (0, 2 or 8 Gy, black bars) by asterisks (*, *p* ≤ 0.05; **, *p* ≤ 0.01; ***, *p* ≤ 0.001) and for multimodal combination (white bar) *vs.* 5-aza-dC + control (dark grey bar) by hash (#, respectively) to clarify (1) the effects compared to irradiation alone and (2) the enhancement of the previously described effect of 5-aza-dC/IR [4]. Additional information about significant synergism/additivity is given in Table 2. Drug concentrations are shown in Table 1

First, we recorded the short-time survival of human MB cells after treatment with single modulators for 6 days using the trypan blue exclusion assay. The rise of vital untreated cells after 6 days compared to initially seeded cells (D283-Med, 16-fold; MEB-Med8a, 41-fold; DAOY, 83-fold) reflects the different growth rates of the three MB cell lines (Fig. 1). Although, short-time cytotoxic effects of the tested drugs may reduce the tumor bulk, some (tumor stem) cells might not be targeted and lead to quick tumor regrowth. Otherwise, cells might grow well in short-term assays but lose their colony-forming ability after a few cell divisions due to the accumulation of DNA damages. Therefore, surviving cells were seeded for clonogenic assays to examine the long-term reproductive survival 10–14 days later (Fig. 2). The plating efficiencies (PE) of untreated cell lines were very similar, between 0.49 ± 0.05 (MEB-Med8a) and 0.56 ± 0.1 (DAOY) (data not shown). From single drug clonogenic assays an inhibitory concentration by which 90 % of clonogenic cells were killed (IC 90) was calculated for each drug (listed in Table 1). If not otherwise noted, these concentrations were used for combination experiments presented individually in Fig. 3 and for joined analysis of all three MB cell lines in Fig. 4. Detailed statistical analysis of overall clonogenic survival is summarized in Table 2.

**Table 2:**
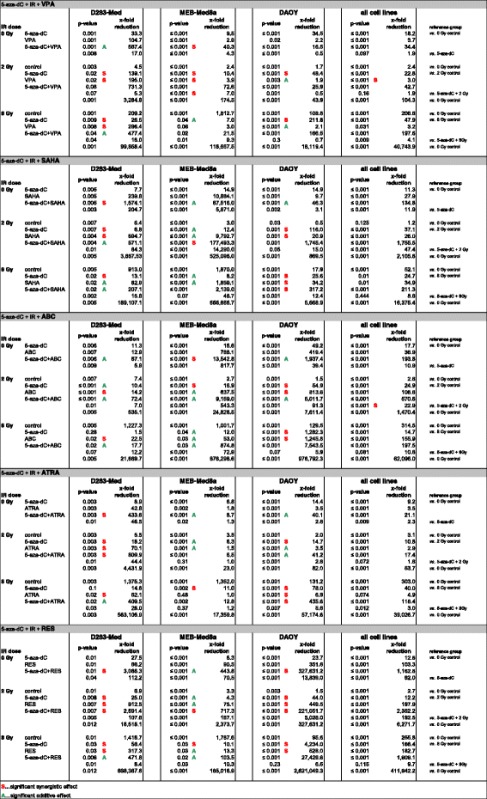
Statistics - Clonogenic survival after combinatorial treatment

*P*-values were calculated by two-tailed, paired Student’s *t*-test in the single cell lines or by non-parametric Wilcoxon rank-sum test in the joint analysis of all cell lines. The x-fold reductions of overall clonogenic survival compared to the indicated reference group are listed. Combinatorial effects conducted with the Bliss independent model are indicated as A = additive or S = synergistic

#### Irradiation (IR)

After IR at 2 Gy, surviving fractions (SF) of clonogenic cells were reduced to 0.17 ± 0.02 (D283-Med, *n* = 5), 0.34 ± 0.02 (MEB-Med8a, *n* = 5), and 0.61 ± 0.04 (DAOY, *n* = 5) (Fig. 3). After IR at 8 Gy, SF was further reduced to 1.63 E-03 ± 7.92 E-04 (D283-Med), 6.76 E-04 ± 8.94 E-05 (MEB-Med8a), and 1.82 E -02 ± 9.45 E-03 (DAOY) (data not shown). The mean SF of all cell lines was 0.37 ± 0.13 at 2 Gy and 0.007 ± 0.006 at 8 Gy.

#### 5-Aza-2′-deoxycytidine (5-aza-dC)

After 6 days of 5-aza-dC treatment, we observed a dose-dependent and cell line-specific decline of short-time survival (Fig. 1a) and long-term clonogenic survival (Fig. 2a) in all three cell lines. Shortening of treatment time to 3 days diminished the inhibitory effect (data not shown). The slowly proliferating D283-Med cells (doubling time DT = 40 h) exhibited the strongest sensitivity (IC 90 = 0.05 μM), whereas the fast growing DAOY cells (DT = 18 h) were most resistant to 5-aza-dC (IC 90 = 0.5 μM). Combination of 5-aza-dC with IR (2 Gy), led to radiosynergistic effects in all cell lines and reduced the overall clonogenic survival by 15-fold (D283-Med, *n* = 5), 8-fold (MEB-Med8a, *n* = 5), and 34-fold (DAOY, *n* = 5) compared to 5-aza-dC alone (Fig. 3).

Joint analysis of all three cell lines revealed a 13-fold reduction of overall clonogenic survival by 5-aza-dC alone (SF = 0.088 ± 0.02; *p* ≤ 0.001) and a 50-fold reduction by 5-aza-dC and 2 Gy (SF = 0.022 ± 0.01; *p* ≤ 0.001) *versus* untreated control (Fig. 4).

#### Valproic acid (VPA)

Relatively high doses of VPA (>1 mM) were required to reduce the short- and long-time survival in all MB cell lines (Figs. 1b and 2b). Combination of 5-aza-dC and VPA showed additive effects of VPA in D283-Med, synergism in MEB-Med8a, but no further reduction in DAOY on overall clonogenic survival compared to 5-aza-dC alone. VPA acted radiosynergistically in D283-Med and MEB-Med8a and radioadditively in DAOY cells. Addition of VPA to 5-aza-dC and IR (2 Gy) led to synergistic effects in MEB-Med8a only, reducing the overall clonogenic survival further by 7-fold (Fig. 3a; significances in Table 2).

Joint analysis of all MB cell lines (*n* = 3) revealed that VPA alone induced a 3.7-fold decrease (SF = 0.27 ± 0.13; *p* ≤ 0.001) and combined with 2 Gy and 5-aza-dC, a 104-fold decrease (SF = 0.01 ± 0.007; *p* ≤ 0.001) of overall clonogenic survival compared to untreated control (Fig. 4a; significances Table 2).

#### Suberanilohydroxamic acid (SAHA)

The maximal tolerated SAHA plasma concentration in humans of 2 μM [37] reduced the relative number of vital cells only by 46–77 % compared to untreated cells (Fig. 1c). In clonogenic assays, the IC 90 was reached only in D283-Med cells. In MEB-Med8a and DAOY cells SAHA (2 μM) resulted in a SF of 0.29 and 0.18 (Fig. 2c). The combination of SAHA and 5-aza-dC revealed synergistic effects in D238-Med and additive effects in MEB-Med8a and DAOY on overall clonogenic survival. Combined with IR, SAHA showed radiosynergistic effects in D283-Med and DAOY and radioadditive effects in MEB-Med8a. In 5-aza-dC/IR-treated cells, SAHA induced additive effects in D283-Med cells and synergistic effects in MEB-Med8a and DAOY cells. Thereby, it further reduced the overall clonogenic survival of 5-aza-dC/2 Gy-treated MB cells by 84-fold (D283-Med), 14,000-fold (MEB-Med8a), and 15-fold (DAOY) (Fig. 3b; significances in Table 2).

Joint analysis of the three MB cell lines revealed a SF of 0.036 ± 0.034 (*p* ≤ 0.001; *n* = 3) in SAHA treated cells. Combination of SAHA with 2 Gy and 5-aza-dC reduced the overall clonogenic survival 2,100-fold compared to untreated control (SF = 4.75 E-04 ± 3.47 E-04; *p* ≤ 0.001; *n* = 3) (Fig. 4b; significances in Table 2).

#### Abacavir (ABC)

The ABC treatment diminished the relative number of vital cells (Fig. 1d) and the overall clonogenic survival (Fig. 2d) in all cell lines with strongest effects on D283 cells. In clonogenic assays, the combination of ABC with 5-aza-dC revealed additive effects in D283-Med and DAOY and synergistic effects in MEB-Med8a. Combined with IR, ABC showed radiosynergistic effects in D283-Med and DAOY and radioadditive effects in MEB-Med8a. In the multimodal treatment with 5-aza-dC and IR, ABC acted additively in all MB cell lines and further reduced the overall clonogenic survival of 5-aza-dC/2 Gy-treated cells by 7-fold (D283-Med), 540-fold (MEB-Med8a), and 90-fold (DAOY) (Fig. 3c; significances Table 2).

Joint analysis of all three MB cell lines revealed a 37-fold reduction of overall clonogenic survival by ABC (SF = 0.027 ± 0.025; *p* ≤ 0.001, *n* = 3). After combination of ABC with 2 Gy and 5-aza-dC, the overall clonogenic survival was diminished 1,470-fold compared to untreated control (SF = 6.80 E-04 ± 5.95 E-04; *p* ≤ 0.001; *n* = 3) (Fig. 4c; significances Table 2).

#### All-trans retinoic acid (ATRA)

ATRA induced differential responses. It reduced dose-dependently the relative vital cell number and surviving fraction (IC 90 = 1.5 μM) in D283-Med cells, whereas in DAOY cells only a moderate response (estimated IC 90 = 10 μM), and in MEB-Med8a cells at concentrations of up to 5 μM no response was induced (Figs. 1e and 2e). Combined with 5-aza-dC, ATRA synergized in D283-Med cells (49-fold reduction *vs.* 5-aza-dC) and showed slight additive effects in DAOY (2.8-fold reduction *vs.* 5-aza-dC) and MEB-Med8a cells (1.3-fold reduction *vs.* 5-aza-dC). Also in combination with IR, ATRA induced radiosynergistic effects in D283-Med and radioadditive effects in MEB-Med8a and DAOY. In the multimodal setting of ATRA with 5-aza-dC and IR, synergistic effects were found in D283-Med and additive effects in DAOY but not in MEB-Med8a cells. Compared to 5-aza-dC/2 Gy-treated cells the overall clonogenic survival was reduced by 44-fold and 2.8-fold, respectively (Fig. 3d; significances Table 2).

Joint analysis of the MB cell lines revealed a 3.5-fold decrease of overall clonogenic survival (SF = 0.28 ± 0.15; *p* ≤ 0.001; *n* = 3) by ATRA alone. The combination of ATRA with 2 Gy and 5-aza-dC showed a 54-fold reduction compared to untreated control cells (SF = 0.019 ± 0.013; *p* ≤ 0.001; *n* = 3) (Fig. 4d; significances Table 2).

#### Resveratrol (RES)

RES concentrations between 0.1–10 μM diminished the relative number of vital cells and clonogenic survival in D283-Med and MEB-Med8a cells, but only slightly affected DAOY cells (Figs. 1f and 2f). Thus, for combination experiments, DAOY cells were treated with 40 μM RES (IC 94), which is the maximum achievable plasma concentration in animal experiments [26]. Combined with 5-aza-dC, RES reduced the overall clonogenic survival of 5-aza-dC-treated cells synergistically in D283-Med (110-fold) and DAOY cells (14,000-fold), and additively in MEB-Med8a cells (70-fold). Also in combination with IR, RES showed radiosynergistic effects in D283-Med and DAOY and radioadditive effects in MEB-Med8a cells. The multimodal setting, combining RES with 5-aza-dC and IR, revealed synergistic action of RES in all cell lines. Thereby, it further reduced the SF of 5-aza-dC/2 Gy-treated cells by 110-fold (D283-Med), 170-fold (MEB-Med8a), and 5,000-fold (DAOY) (Fig. 3e; significances Table 2).

Joint analyses of all three cell lines showed a 100-fold reduction of overall clonogenic survival by RES alone (SF = 0.01 ± 0.004; *p* ≤ 0.001). Combined with 2 Gy and 5-aza-dC, the overall clonogenic survival was reduced 6,350-fold compared to untreated cells (SF = 1.59 E-04 ± 1.32 E-04; *p* ≤ 0.001; *n* = 3) (Fig. 4e; significances Table 2).

### Mechanistic studies of IR, 5-aza-dC, RES, and ABC in tumor cells

The combination of IR and 5-aza-dC with RES or ABC reduced the clonogenic survival of human MB cells most efficiently compared to any other single or combined drug treatment tested. To reveal the mechanism of action we analyzed apoptosis, autophagy, cell cycle distribution, and DNA double-strand break repair after these most promising treatments using the cell line specific IC 90 of of 5-aza-dC, RES, or ABC (see Table 1) and IR at 2 Gy.

#### Vital cell count, Apoptosis, and Autophagy

**Fig. 5:**
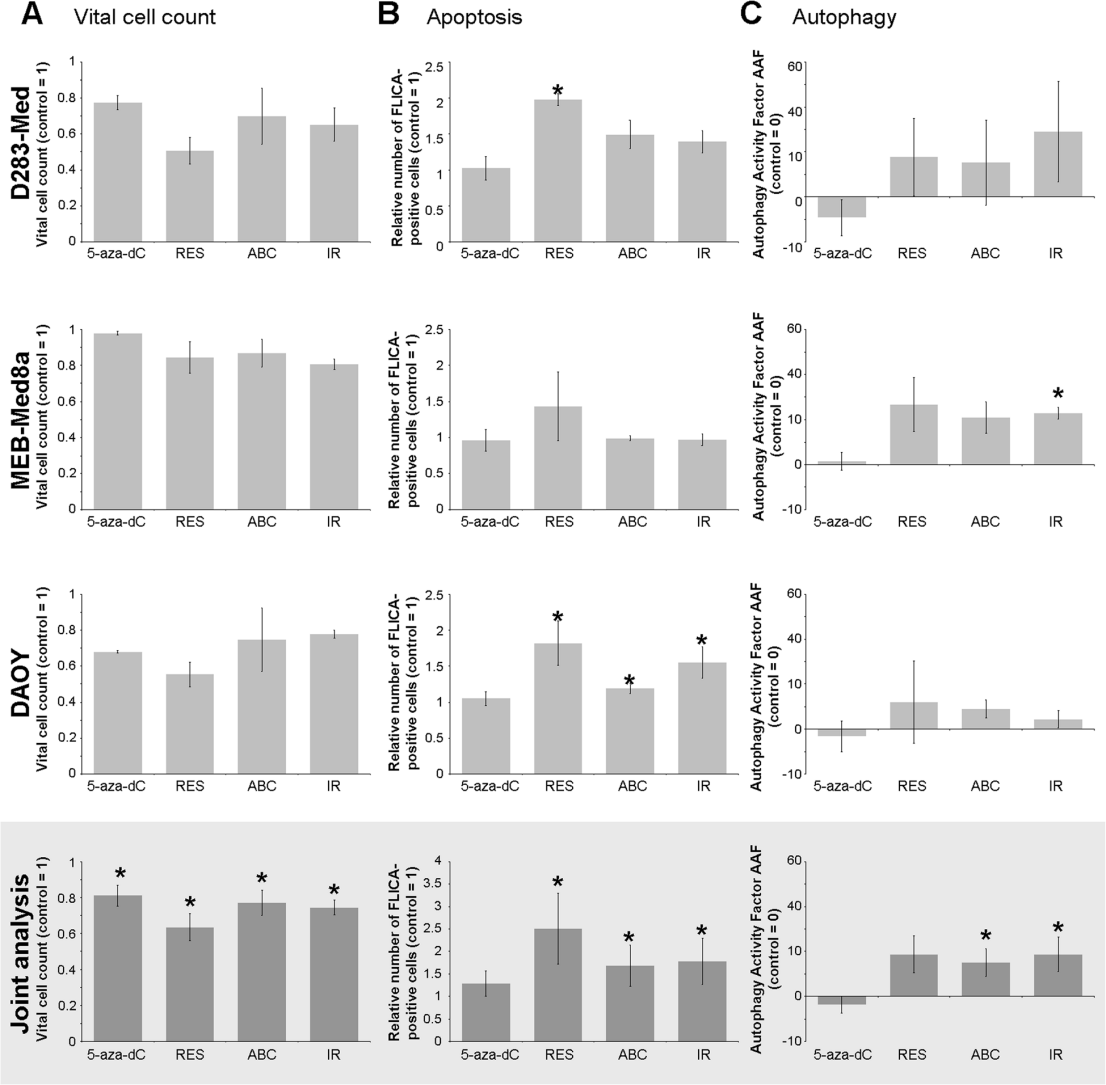
Vital cell count (**a**), Apoptosis (**b**), and Autophagy (**c**). Vital cell count was determined by trypan blue exclusion assay, apoptosis by FAM-FLICA™ in vitro Caspase 3/7 Detection Kit, and autophagy by Cyto-ID® Autophagy Detection Kit, 18 h after treatment with the single drugs (5-aza-dC, RES, ABC; concentrations see Table 1) or irradiation with 2 Gy. Data were normalized to the untreated control group and presented as mean ± SEM, *n* = 3. Statistical significances are presented *vs.* control by asterisks (*, *p* ≤ 0.05; **, *p* ≤ 0.01). The joint analysis included the relative data of the three MB cell lines

The joint analysis of all three MB cell lines revealed a significant reduction of vital cell count 18 h after IR (2 Gy) or treatment with 5-aza-dC, RES, or ABC by 25, 19, 37, or 23 %, respectively (*n* = 3, *p* ≤ 0.05. Fig. 5a). Responses were similar in all three cell lines, except for DAOY cells being the least sensitive for ABC and IR treatments.

**Fig. 6:**
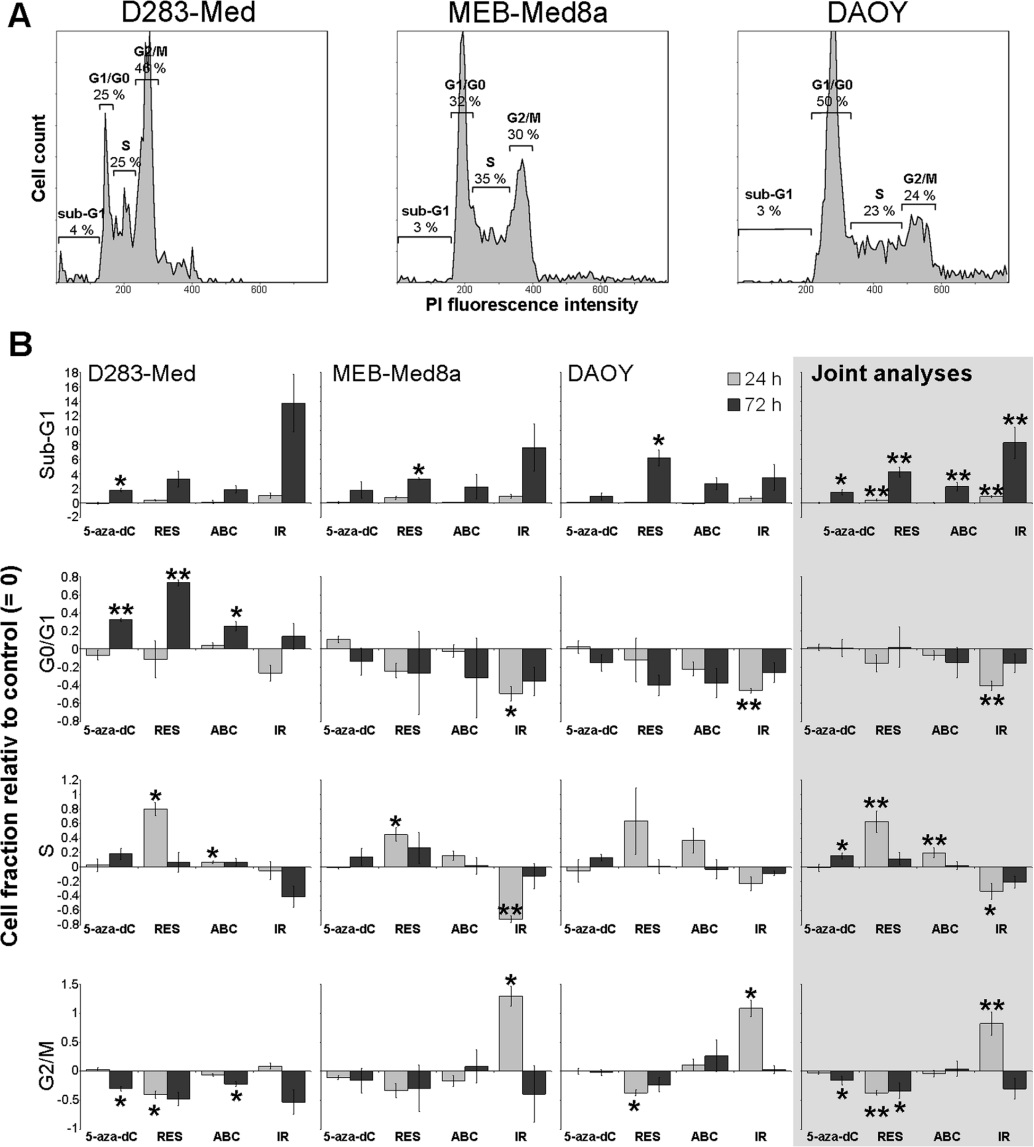
Cell cycle distribution. 24 h and 72 h after treatment with single drugs (5-aza-dC, RES, ABC; concentrations see Table 1) or irradiation with 2 Gy, cells were fixed and stained with propidium iodide (PI). The fluorescence intensity of the PI-positive DNA correlates with the cell cycle phase distribution and was measured by flow cytometry. **a** Histograms of the cell cycle distribution in untreated control cells. The different cell cycle phases corresponding to the PI fluorescence intensity are indicated. **b** Relative fraction of cells distributed in sub-G1, G0/G1, S, or G2/M cell cycle phase, grey bars: 24 h, black bars: 72 h after drug administration or IR. Values are normalized to control (=0) and presented as mean ± SEM, *n* = 3. Statistical significances are presented *vs.* control by asterisks (*, *p* ≤ 0.05; **, *p* ≤ 0.01; ***, *p* ≤ 0.001). The joint analysis included the relative data of the three MB cell lines

Treatments led also to a significant increase of apoptotic cells in caspase 3/7 induction assays (Fig. 5b) and sub-G1 phase quantification (Fig. 6b) in joint analyses (*n* = 3, *p* ≤ 0.05 to *p* ≤ 0.01). The strongest effects on caspase 3/7 induction (18 h) and on sub-G1 apoptotic cell fraction (24/72 h) were observed after RES treatment and IR resulting in a significant 2.5-fold and 1.8-fold enhancement of caspase 3/7, respectively. This was followed by a 5-fold and 9-fold increase of sub-G1 cells 72 h after RES treatment and IR. Single cell line analysis revealed that MEB-Med 8a cells were most resistant showing no significant induction of caspase 3/7, although a significant enrichment of sub-G1 cells 72 h after RES could be observed.

Autolysosome staining to assess autophagy induction showed significant enhancements only after ABC or IR treatment compared to control cells (joint analysis, *n* = 3, *p* ≤ 0.05, Fig. 5c). Most pronounced effects were detected in MEB-Med 8a cells (29-fold after IR, *p* ≤ 0.05).

#### Cell cycle distribution

Joint analyses of the three MB cell lines revealed a significant increase of sub-G1 apoptotic cell population in all treatment groups after 72 h (*n* = 3, *p* ≤ 0.05 to ≤ 0.01). This effect was most pronounced after IR (2 Gy) and RES treatment (Fig. 6b). 5-Aza-dC (72 h) and RES (24 h) additionally induced a significant enriched S phase fraction with a concomitant drop in G2/M cell fraction. Similarly, 24 h after treatment with ABC, a significant enrichment of S phase cells could be observed, though without an impact on the G1 or G2/M cell fraction. Only IR caused a significant G2/M arrest.

Within the cell lines, D283-Med cells showed the strongest response, all single drugs induced a G1 arrest accompanied by a decrease of G2/M cell fraction 72 h after treatment.

#### DNA double-strand breaks

**Fig. 7:**
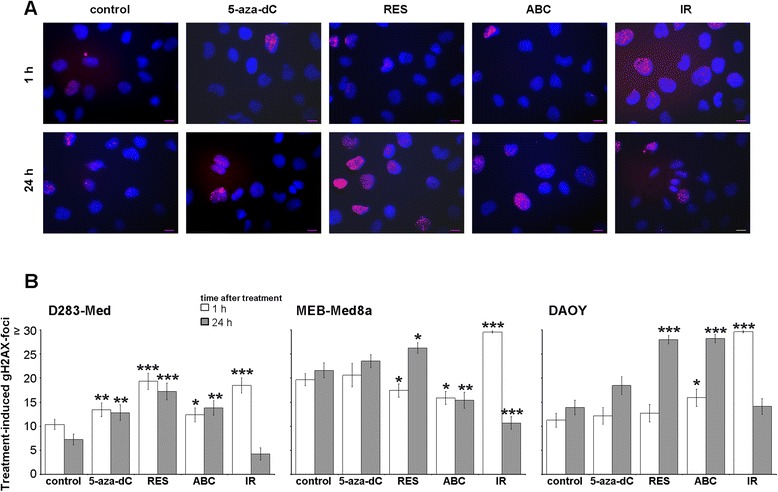
Detection of DNA double-strand breaks (DSB). The DSB repair protein gH2AX was quantified by immune fluorescence microscopy after 1 h and 24 h treatment with the single drugs (5-aza-dC, RES, ABC; concentrations see Table 1) or 1 h and 24 h after irradiation with 2 Gy. **a** Representative images of gH2AX (red) and DAPI (blue) staining in DAOY cells; scale bar = 12 μm. **b** Treatment-induced gH2AX-foci relative to control cells in the single MB cell lines. At least 50 nuclei were counted with a maximum of 30 foci per cell. Experiment was performed once and data are presented as mean ± SEM. Statistical significances are presented *vs.* control by asterisks (*, *p* ≤ 0.05; **, *p* ≤ 0.01; ***, *p* ≤ 0.001)

The IR-induced G2/M arrest goes along with significant enhancement of gH2AX DNA repair protein expression 1 h after IR (2 Gy) in all three MB cells (Fig. 7). The number of gH2AX foci felt below control level after 24 h indicating complete repair of IR-induced DSBs in surviving cells. Also RES and ABC are able to induce gH2AX foci at least in two of the three cell lines, whereas 5-aza-dC induced the formation of gH2AX foci only in D283-Med cells.

### Neurotoxic studies in neural progenitor cells

**Fig. 8:**
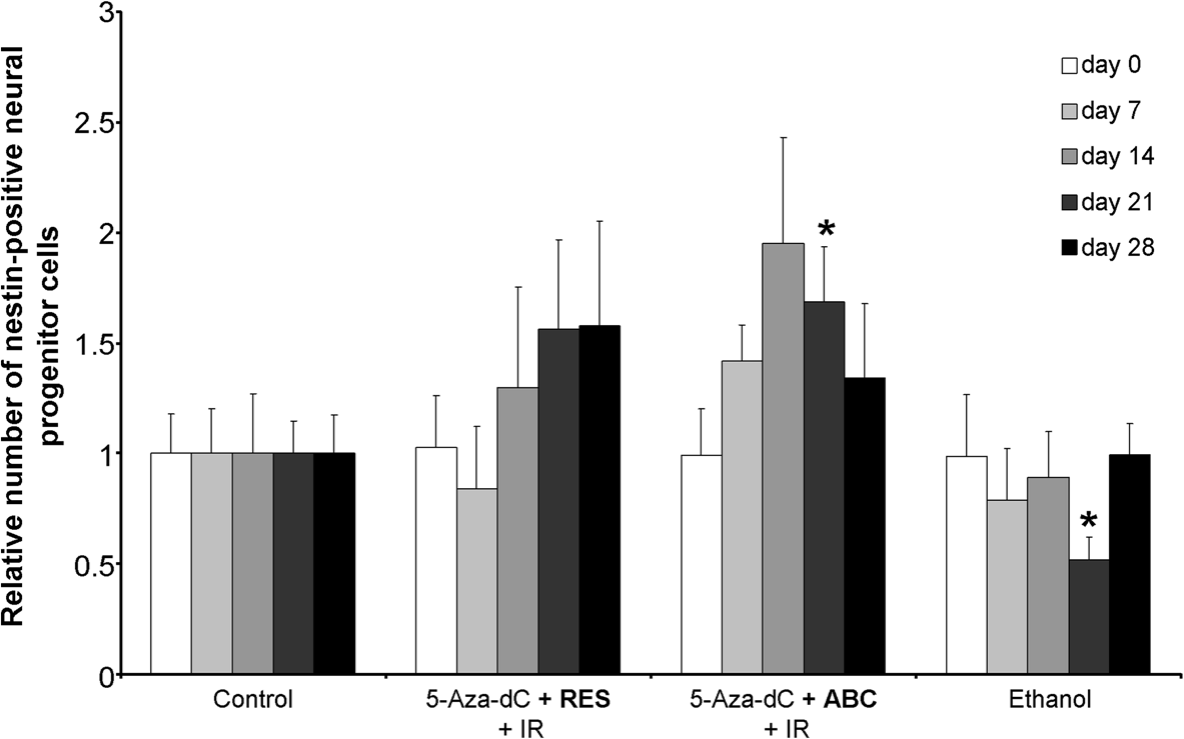
Life imaging analysis of neural progenitor cell number in murine hippocampal tissue slice cultures. The number of nestin-positive cells is shown at day 0, 7, 14, 21, and 28 after IR (8 Gy) or combinatorial treatment with 5-aza-dC, IR and RES or ABC. Ethanol control was included as RES was dissolved in ethanol. Data are normalized to the untreated control group (=1). Results are presented as mean ± SEM; *, *p* ≤ 0.05; *n* = 6 compared to untreated control

Nestin-positive neural progenitors were analyzed by life-imaging microscopy of cultured murine hippocampal slices from transgenic nestin-CFPnuc C57BL/J6 mice to examine potential neurotoxic effects of the most promising multimodal approach (5-aza-dC + IR and RES or ABC) (Fig. 8). To detect short- and long-term effects, analysis were conducted at day 7, 14, 21 and 28 after treatment start. Administration of 5-aza-dC and IR combined with RES or ABC did not reduce the nestin-positive neural progenitor cell pool compared to untreated control.

## Discussion

Despite of surgery, irradiation, and chemotherapy, some remaining therapy-resistant tumor cells may lead to tumor relapse. Here, we follow the hypothesis that tumor control might be enhanced by a multimodal treatment approach, exploiting some of the hallmarks of cancer (detailed in [38]). Based on the positive results of our previous studies investigating the effects of IR and 5-aza-dC [4], and of 5-aza-dC with additional modulators [18], we investigated here for the first time the effect of a triple combination: IR, 5-aza-dC and one additional epigenetic modifier (SAHA, VPA) or differentiation-inducer (RES, ABC and ATRA).

To assess the impact of each treatment component, we examined single and combinatorial effects on short-term survival and long-term reproductive survival in three human MB cell lines. We emphasize that all drug concentrations used here, except of VPA and ABC, are within the range achievable in human or animal plasma [23–28].

### Irradiation

In accordance to our previous results [18], the MB cell line with the slowest growth rate (D283-Med) was found to be most sensitive to IR, whereas the one with the fastest growth rate (DAOY) was the least sensitive concerning the reduction of short-term vitality, enrichment of apoptotic sub-G1 cells, and also of long-term reproductive survival. Although a higher amount of untreated D283-Med cells is located in the radiosensitive G2/M cell cycle phase compared to DAOY cells which exhibited a higher amount of radioresistant G1/G0 cells, more DSBs were induced in the DAOY then in D283-Med cells. We hypothesize that the p53-wild-type D283-Med cells may prefer non-homologous end-joining (NHEJ) as the main DNA repair pathway which is usually executed within minutes after IR (reviewed in [39]) and, therefore, it is probably not seen 1 h after IR. As NHEJ is more error-prone than the DSB repair by homologue recombination (HR), this might explain the enhanced sub-G1 apoptotic cell fraction observed in D238-Med. Nevertheless, DSB repair was completed 24 h after IR in the surviving cells of both cell lines. Our results show, that the induction of gH2AX foci by IR is accompanied with an enhanced G2/M arrest in p53-mutant DAOY cells. This is in line with findings in other tumor cells [40]. Additionally, we observed that IR induces autophagy in human MB cells. It is already known that IR may induce autophagy as a rescue mechanism resulting in radioresistance but also as cytotoxic process resulting in cell death (reviewed in [41, 42]). However, we revealed that autophagy did not account for the long-term radiosensitivity of clonogenic MB cells indicating that other mechanisms such as DSB repair efficiency, suggested by the results above, may play a role here.

The multimodal treatments with 8 Gy IR generally support the findings after IR with 2 Gy (Table 2). Fewer significances found for combinatorial treatments are possibly due to the much lower overall surviving fraction after 8 Gy IR compared with 2 Gy IR.

### 5-Aza-2′-deoxycytidine (5-aza-dC)

The 5-aza-dC-induced enhancement of mortality, inhibition of proliferation, and reduction of clonogenic survival in all three MB cell lines confirm our previous data [4]. Both, its ability to form DNA enzyme adducts (5-aza-dC/DNMT) inhibiting cell cycle progression and also leading to DSB as well as its function as *de novo* methyltransferase inhibitor (DNMTi) [43] are thought to contribute to these anti-cancer effects. The lack of 5-aza-dC-induced apoptosis and autophagy demonstrated here goes along with the only weak induction of gH2AX foci, mainly seen in D283-Med cells. Therefore, we assume that the 5-aza-dC-induced reduction of the short-term and long-term survival in MB cells might be rather caused by its inhibition of proliferation and/or epigenetic properties [4, 44, 45].

We demonstrated a radiosynergistic reduction of overall clonogenic survival after combining 5-aza-dC with IR. By modulation of the former treatment schedule (5-aza-dC only prior to IR) to 5-aza-dC treatment prior and after IR, we could improve the previously found radioadditive effect of 5-aza-dC [4]. This 5-aza-dC-induced enhancement of radiosensitivity has also been described in other tumor entities [46–49]. It has been suggested by others that the induction of a G2/M arrest by 5-aza-dC treatment prior to IR may account at least partly for its radiosensitizing effect [48]. This could not be confirmed by our findings. Joint analysis of our MB cell lines demonstrated a slightly prolonged DNA synthesis cell cycle phase possibly resulting in the reduced G2/M fraction, after 24 h 5-aza-dC treatment. Additional mechanisms like the p53-dependent induction of senescence demonstrated in hepatocellular carcinoma [50] might play a role here and also could explain the increased sensitivity of *p53*-wild-type D283-Med and MEB-Med8a cells compared to *p53*-mutated DAOY cells. Similarly, in experiments with colon cancer cells, Karpf et al. revealed that 5-aza-dC is more effective to mediate growth arrest and cytotoxicity in *p53*-wild-type *versus p53*-mutated cells [51]. These observations indicate that the treatment of MB patients with *p53* wild-type tumors (the majority of patients) might require lower 5-aza-dC concentrations than the one with *p53*-mutated tumors.

### Valproic acid (VPA)

VPA, a histone deacetylase inhibitor (HDACi) [52] and a replication-independent DNMTi [53] induced a moderate decline in relative vital MB cell number and clonogenic survival, which goes along with our previous findings [18], and supports its anti-tumor effects reported in other tumor models [54, 55].

VPA induces apoptosis and growth arrest in MB cells by a p53-independent mechanism through alteration of histone acetylation and thereby gene and protein expression [52]. Indeed, all three tested MB cell lines showed largely similar sensitivities to VPA alone. The combination of VPA and 5-aza-dC showed differential effects on MB cell overall clonogenic survival and the previously measured metabolic activity [18]. After treatment, an enhanced metabolic activity but a reduced clonogenic potential was found in MEB-Med8a cells. In DAOY cells however, despite of reduced metabolic activity, the clonogenicity was slightly enhanced. These results emphasize the importance of long-term reproductive survival assays to evaluate the clonogenic potential of tumor stem cells after different treatment regimes. The radiosynergistic effect of VPA on clonogenic survival, demonstrated in two cell lines here, goes along with findings in glioma cell lines and in a glioma xenograft mouse model, where an inhibition of radiation-induced DSB repair by VPA has been suggested [56]. Our results underline the potential of VPA in combination with radio- and chemotherapy, recently demonstrated in retrospective studies of glioblastoma patients [57, 58].

### Suberanilohydroxamic acid (SAHA)

The HDACi SAHA alone only slightly reduced the clonogenic survival despite of its strong effect on the metabolic activity published previously [18]. Interestingly, addition of SAHA significantly diminished the 5-aza-dC-induced reduction of overall clonogenic survival in all MB cell lines. Strong additive effects of 5-aza-dC and SAHA are described also by others in leukemic and ovarian cancer cell lines and are thought to be transmitted through the induction of cell cycle arrest (G1/S by SAHA; G2/M by 5-aza-dC) and apoptosis [59, 60].

The combination of SAHA with IR showed radiosynergistic effects on overall clonogenic survival in all tested MB cell lines, even slightly stronger than found with VPA. Similarly, other groups observed a radiosensitizing action of SAHA on osteosarcoma and atypical teratoid rhabdoid tumor cells in vitro and in a rhabdoid tumor mouse model in vivo [61, 62]. Regarding 5-aza-dC-treated irradiated MB cells, SAHA induced synergistic effects on overall clonogenic survival in all three tested MB cell lines and in this combination seems to be more effective than the HDACi VPA.

### Abacavir (ABC)

ABC has been shown to down-regulate the telomerase activity, which is overexpressed in the vast majority of cancers including MB [63]. Sparing normal brain cells due to their low or even not existing telomerase activity, the inhibition of telomerase in brain tumor cells seems to be a promising therapeutic strategy for MB. ABC alone decreased the overall clonogenic survival in all MB cell lines. The ABC-induced reduction of vital cell count goes along with its induction of apoptosis and autophagy. Importantly, we found that ABC acts strongly additive with 5-aza-dC on the reduction of the overall clonogenic survival in all three MB cell lines. It seems worthwhile to investigate this combination in animal models to clarify if it could be an alternative therapy option for infants (<4 years), who cannot obtain radiotherapy due to unwanted adverse effects.

Our experiments revealed also radiosynergistic (DAOY, D283-Med) and radioadditive (MEB-Med8a) effects of ABC on the overall clonogenic survival. In accordance, it has been shown that the inhibition of telomerase activity can sensitize tumor cells to irradiation [64, 65], partly mediated by prolongation of DSB repair. In two-out-of-three cell lines we found indeed a higher amount of gH2AX foci after 24 h ABC treatment compared to control. MEB-Med8a behaved differentially possibly due to a high amount of initial (control) DSB. It has been suggested that the upregulation of telomerase activity in tumor cells may represent a protective mechanism against DNA damage [65]. This hypothesis is supported by Akiyama et al., who showed that the overexpression of telomerase protects leukemia cells from DSB-triggered apoptosis [66].

The ABC-induced significant reduction of overall clonogenic survival in all 5-aza-dC/IR-treated MB cell lines indicated a therapeutic potential of the triple combination.

### All-trans retinoic acid (ATRA)

ATRA, the prototype of differentiation therapy, induced differential responses in the three MB cell lines. Similar to our previous studies on metabolic activity [18], the strongest effect on overall clonogenic survival was found in the OTX2-expressing D283-Med cell line, supporting recent findings of OTX2 as a medulloblastoma oncogene and possible target for ATRA [67]. MEB-Med8a cells seemed to be ATRA-resistant, whereas in DAOY cells a moderate effect was seen. In accordance, an ATRA-mediated induction of caspase 3 followed by inhibition of proliferation and apoptosis was reported in D283-Med cells and to a lesser extend in DAOY cells [68]. Also, enhancement of OTX2 (orthodenticle homeobox 2) protein levels, involved in ATRA-mediated tumor cell death, has been shown in D283-Med but not in DAOY cells [67].

By combination of ATRA and 5-aza-dC as well as ATRA and IR, we showed synergism in D283-Med (1.5 μM), a moderate reduction of clonogenicity in DAOY and also in the ATRA-resistant MEB-Med8a cell line, although at relatively high doses (10 μM) only. We hypothesize that this synergistic effect could be caused by 5-aza-dC- induced demethylation of the *CRABP-II* gene as suggested by Fu et al. [69]. Therefore, ATRA, if combined with demethylating agents, might be an interesting substance to treat MB tumor stem cells to bypass their radio- and chemoresistance.

### Resveratrol

The plant polyphenol RES exhibits tumor-preventive as well as anticancer effects depending on concentration, cell type, and microenvironment [21, 70–72]. Studies in MB cells have shown the induction of cell cycle arrest, apoptosis and neuronal differentiation by RES [73–75]. In accordance, we could show a RES-mediated prolongation of DNA synthesis cell cycle phase with an increase of gH2AX foci formation. This is followed by the reduction of vital cell count partially due to the induction of apoptosis. Our previous experiments revealed synergistic effects with 5-aza-dC on the metabolic activity of human MB cell lines [18]. Our recent data from clonogenic survival experiments confirm an anti-cancer effect of RES combined with 5-aza-dC and/or IR in all three MB cell lines. Although RES has been shown to prevent normal cells from treatment-induced damage [18, 72, 76, 77], used on its own, it seems to induce adverse effects [78].

It is of special interest that combinatorial treatments with RES reduced the overall clonogenic survival most strongly in the *p53*-mutated DAOY cell line. Numerous clinical studies confirmed the unfavorable prognosis of patients with *p53*-mutated MBs (about 10 % of all MB patients); they all died in the first 5 years after diagnosis [79–83]. Animal experiments are warranted to reveal if RES might be an interesting adjuvant therapy option for high-risk patients with p53-mutated tumors.

### Neurotoxicity of 5-aza-dC, RES, and ABC

This is the first report showing data concerning the neurotoxicity of a multimodal anti-tumor treatment approach with 5-aza-dC, IR, and RES or ABC using a murine hippocampal tissue slice model. Thereby, no toxic effects on neural progenitor cells were observed. Park et al. revealed adverse effects of RES alone on the murine hippocampal neurogenesis and cognitive function in vivo and in vitro [78]. However, in accordance to our observations, Denissova et al. [84] showed that RES enhances the survival of irradiated mouse embryonic stem cells by accelerating and refining radiation-induced DNA repair.

The nucleoside analogue ABC is known to have a high potential risk of neuronal damage in primary cell cultures of rat forbrain [85]. Nevertheless, in our murine ex vivo hippocamal tissue slice model we could not detect any significant neurotoxicity of ABC in combination with 5-aza-dC and IR regarding nestin-positive neural precursor cells.

## Conclusion

The differential results of short- and long-term survival in these and also metabolic tests in previous studies [18] underline the importance of clonogenic assays. Successful antitumor therapy may benefit from personalized medicine reflected here in the case of ATRA, working best in selected OTX2 expressing samples. Nevertheless, the triple combination of 5-aza-dC/IR and ABC or RES turned out to work effectively in all three cell lines warranting further in vivo investigations.

Long-term analysis of neural stem cell numbers in a murine hippocampal slice culture model revealed no neurotoxic potential for these drug combinations. Also VPA and SAHA reduced the reproductive survival of 5-aza-dC/IR treatments further but to a lower extend.

The most powerful combinations of our in vitro experiments warrant in vivo experiments in an orthotopic MB mouse model. If successfully translated in vivo, especially the combination experiments without IR might provide adjuvant alternative therapy strategies in infants (<4 years).

## Abbreviations

5-aza-dC, 5-aza-2′-deoxycytidine; ABC, abacavir; ATRA, all-*trans* retinoic acid; DNMTi, *de novo* methyltransferase inhibitor; DSB, DNA double-strand breaks; HDACi, histone deacetylase inhibitor; IR, irradiation; MB, medulloblastoma; PE, plating efficiency; RES, resveratrol; SAHA, suberoylanilide hydroxamic acid; SF, surviving fraction; TSG, tumor suppressor gene; VPA, valproic acid.

## References

[1] Rickert CH, Paulus W (2001). Epidemiology of central nervous system tumors in childhood and adolescence based on the new WHO classification. Childs Nerv Syst.

[2] Gibson P, Tong Y, Robinson G, Thompson MC, Currle DS, Eden C (2010). Subtypes of medulloblastoma have distinct developmental origins. Nature.

[3] de Bont JM, Packer RJ, Michiels EM, den Boer ML, Pieters R (2008). Biological background of pediatric medulloblastoma and ependymoma: a review from a translational research perspective. Neuro Oncol.

[4] Patties I, Jahns J, Hildebrandt G, Kortmann RD, Glasow A (2009). Additive effects of 5-aza-2′-deoxycytidine and irradiation on clonogenic survival of human medulloblastoma cell lines. Strahlenther Onkol.

[5] Christman JK (2002). 5-Azacytidine and 5-aza-2′-deoxycytidine as inhibitors of DNA methylation: mechanistic studies and their implications for cancer therapy. Oncogene.

[6] Al-Romaih K, Somers GR, Bayani J, Hughes S, Prasad M, Cutz JC (2007). Modulation by decitabine of gene expression and growth of osteosarcoma U2OS cells in vitro and in xenografts: identification of apoptotic genes as targets for demethylation. Cancer Cell Int.

[7] Gu S, Tian Y, Chlenski A, Salwen HR, Lu Z, Raj JU (2012). Valproic acid shows a potent antitumor effect with alteration of DNA methylation in neuroblastoma. Anticancer Drugs.

[8] Peart MJ, Smyth GK, van Laar RK, Bowtell DD, Richon VM, Marks PA (2005). Identification and functional significance of genes regulated by structurally different histone deacetylase inhibitors. Proc Natl Acad Sci U S A.

[9] Shin DY, Sung KH, Kim GY, Kim WJ, Yoo YH, Choi YH (2013). Decitabine, a DNA methyltransferases inhibitor, induces cell cycle arrest at G2/M phase through p53-independent pathway in human cancer cells. Biomed Pharmacother.

[10] Han BR, You BR, Park WH (2013). Valproic acid inhibits the growth of HeLa cervical cancer cells via caspase-dependent apoptosis. Oncol Rep.

[11] Wu Z, Ma C, Shan Z, Ju Y, Zhao Q. Histone deacetylase inhibitors suppress the growth of human osteosarcomas in vitro and in vivo. J.BUON. 2013;18:1032-7. 24344034

[12] Pawlik TM, Keyomarsi K (2004). Role of cell cycle in mediating sensitivity to radiotherapy. Int J Radiat Oncol Biol Phys.

[13] Palmer JO, Kasselberg AG, Netsky MG (1981). Differentiation of Medulloblastoma. Studies including immunohistochemical localization of glial fibrillary acidic protein. J Neurosurg.

[14] Burger PC, Grahmann FC, Bliestle A, Kleihues P (1987). Differentiation in the medulloblastoma. A histological and immunohistochemical study. Acta Neuropathol.

[15] Singh SK, Hawkins C, Clarke ID, Squire JA, Bayani J, Hide T (2004). Identification of human brain tumour initiating cells. Nature.

[16] Eyler CE, Rich JN (2008). Survival of the fittest: cancer stem cells in therapeutic resistance and angiogenesis. J Clin Oncol.

[17] Al-Hajj M, Clarke MF (2004). Self-renewal and solid tumor stem cells. Oncogene.

[18] Patties I, Kortmann RD, Glasow A (2013). Inhibitory effects of epigenetic modulators and differentiation inducers on human medulloblastoma cell lines. J Exp Clin Cancer Res.

[19] Bai R, Siu IM, Tyler BM, Staedtke V, Gallia GL, Riggins GJ (2010). Evaluation of retinoic acid therapy for OTX2-positive medulloblastomas. Neuro Oncol.

[20] Chang Q, Pang JC, Li J, Hu L, Kong X, Ng HK (2004). Molecular analysis of PinX1 in medulloblastomas. Int J Cancer.

[21] Muqbil I, Beck FW, Bao B, Sarkar FH, Mohammad RM, Hadi SM (2012). Old wine in a new bottle: the Warburg effect and anticancer mechanisms of resveratrol. Curr Pharm Des.

[22] Andres-Mach M, Rola R, Fike JR (2008). Radiation effects on neural precursor cells in the dentate gyrus. Cell Tissue Res.

[23] Karahoca M, Momparler RL (2013). Pharmacokinetic and pharmacodynamic analysis of 5-aza-2′-deoxycytidine (decitabine) in the design of its dose-schedule for cancer therapy. Clin Epigenetics.

[24] Ramalingam SS, Kummar S, Sarantopoulos J, Shibata S, LoRusso P, Yerk M (2010). Phase I study of vorinostat in patients with advanced solid tumors and hepatic dysfunction: a National Cancer Institute Organ Dysfunction Working Group study. J Clin Oncol.

[25] Lefebvre P, Thomas G, Gourmel B, Agadir A, Castaigne S, Dreux C (1991). Pharmacokinetics of oral all-trans retinoic acid in patients with acute promyelocytic leukemia. Leukemia.

[26] Asensi M, Medina I, Ortega A, Carretero J, Bano MC, Obrador E (2002). Inhibition of cancer growth by resveratrol is related to its low bioavailability. Free Radic Biol Med.

[27] Atmaca A, Al-Batran SE, Maurer A, Neumann A, Heinzel T, Hentsch B (2007). Valproic acid (VPA) in patients with refractory advanced cancer: a dose escalating phase I clinical trial. Br J Cancer.

[28] Weller S, Radomski KM, Lou Y, Stein DS (2000). Population pharmacokinetics and pharmacodynamic modeling of abacavir (1592U89) from a dose-ranging, double-blind, randomized monotherapy trial with human immunodeficiency virus-infected subjects. Antimicrob Agents Chemother.

[29] Petitjean A, Mathe E, Kato S, Ishioka C, Tavtigian SV, Hainaut P (2007). Impact of mutant p53 functional properties on TP53 mutation patterns and tumor phenotype: lessons from recent developments in the IARC TP53 database. Hum Mutat.

[30] Zhukova N, Ramaswamy V, Remke M, Martin DC, Castelo-Branco P, Zhang CH (2014). WNT activation by lithium abrogates TP53 mutation associated radiation resistance in medulloblastoma. Acta Neuropathol Commun.

[31] Encinas JM, Vaahtokari A, Enikolopov G (2006). Fluoxetine targets early progenitor cells in the adult brain. Proc Natl Acad Sci U S A.

[32] Gahwiler BH, Capogna M, Debanne D, McKinney RA, Thompson SM (1997). Organotypic slice cultures: a technique has come of age. Trends Neurosci.

[33] Kluge A, Hailer NP, Horvath TL, Bechmann I, Nitsch R (1998). Tracing of the entorhinal-hippocampal pathway in vitro. Hippocampus.

[34] Lendahl U, Zimmerman LB, McKay RD (1990). CNS stem cells express a new class of intermediate filament protein. Cell.

[35] BLISS CI (1939). THE TOXICITY OF POISONS APPLIED JOINTLY1. Ann Appl Biol.

[36] Prichard MN, Shipman C (1990). A three-dimensional model to analyze drug-drug interactions. Antiviral Res.

[37] Ramalingam SS, Maitland ML, Frankel P, Argiris AE, Koczywas M, Gitlitz B (2010). Carboplatin and Paclitaxel in combination with either vorinostat or placebo for first-line therapy of advanced non-small-cell lung cancer. J Clin Oncol.

[38] Hanahan D, Weinberg RA (2011). Hallmarks of cancer: the next generation. Cell.

[39] Kakarougkas A, Jeggo PA (2014). DNA DSB repair pathway choice: an orchestrated handover mechanism. Br J Radiol.

[40] Borst GR, McLaughlin M, Kyula JN, Neijenhuis S, Khan A, Good J (2013). Targeted radiosensitization by the Chk1 inhibitor SAR-020106. Int J Radiat Oncol Biol Phys.

[41] Yang Y, Yang Y, Yang X, Zhu H, Guo Q, Chen X (2015). Autophagy and its function in radiosensitivity. Tumour Biol.

[42] Ondrej M, Cechakova L, Durisova K, Pejchal J, Tichy A: To live or let die: Unclear task of autophagy in the radiosensitization battle. Radiother Oncol 2016;119:265-75. 10.1016/j.radonc.2016.02.028 26993419

[43] Palii SS, Van Emburgh BO, Sankpal UT, Brown KD, Robertson KD (2008). DNA methylation inhibitor 5-Aza-2′-deoxycytidine induces reversible genome-wide DNA damage that is distinctly influenced by DNA methyltransferases 1 and 3B. Mol Cell Biol.

[44] Harada K, Toyooka S, Shivapurkar N, Maitra A, Reddy JL, Matta H (2002). Deregulation of caspase 8 and 10 expression in pediatric tumors and cell lines. Cancer Res.

[45] Kongkham PN, Northcott PA, Croul SE, Smith CA, Taylor MD, Rutka JT (2010). The SFRP family of WNT inhibitors function as novel tumor suppressor genes epigenetically silenced in medulloblastoma. Oncogene.

[46] Li Y, Geng P, Jiang W, Wang Y, Yao J, Lin X (2014). Enhancement of radiosensitivity by 5-Aza-CdR through activation of G2/M checkpoint response and apoptosis in osteosarcoma cells. Tumour Biol.

[47] Wang H, Chen P, Wang J, Santhanam R, Aimiuwu J, Saradhi UV (2013). In vivo quantification of active decitabine-triphosphate metabolite: a novel pharmacoanalytical endpoint for optimization of hypomethylating therapy in acute myeloid leukemia. AAPS J.

[48] De SH, Kimpe M, Isebaert S, Nuyts S (2009). A systematic assessment of radiation dose enhancement by 5-Aza-2′-deoxycytidine and histone deacetylase inhibitors in head-and-neck squamous cell carcinoma. Int J Radiat Oncol Biol Phys.

[49] Fattet S, Haberler C, Legoix P, Varlet P, Lellouch-Tubiana A, Lair S (2009). Beta-catenin status in paediatric medulloblastomas: correlation of immunohistochemical expression with mutational status, genetic profiles, and clinical characteristics. J Pathol.

[50] Venturelli S, Berger A, Weiland T, Essmann F, Waibel M, Nuebling T (2013). Differential induction of apoptosis and senescence by the DNA methyltransferase inhibitors 5-azacytidine and 5-aza-2′-deoxycytidine in solid tumor cells. Mol Cancer Ther.

[51] Karpf AR, Moore BC, Ririe TO, Jones DA (2001). Activation of the p53 DNA damage response pathway after inhibition of DNA methyltransferase by 5-aza-2′-deoxycytidine. Mol Pharmacol.

[52] Li XN, Shu Q, Su JM, Perlaky L, Blaney SM, Lau CC (2005). Valproic acid induces growth arrest, apoptosis, and senescence in medulloblastomas by increasing histone hyperacetylation and regulating expression of p21Cip1, CDK4, and CMYC. Mol Cancer Ther.

[53] Detich N, Bovenzi V, Szyf M (2003). Valproate induces replication-independent active DNA demethylation. J Biol Chem.

[54] Bruzzese F, Leone A, Rocco M, Carbone C, Piro G, Caraglia M (2011). HDAC inhibitor vorinostat enhances the antitumor effect of gefitinib in squamous cell carcinoma of head and neck by modulating ErbB receptor expression and reverting EMT. J Cell Physiol.

[55] Neri P, Tagliaferri P, Di Martino MT, Calimeri T, Amodio N, Bulotta A (2008). In vivo anti-myeloma activity and modulation of gene expression profile induced by valproic acid, a histone deacetylase inhibitor. Br J Haematol.

[56] Camphausen K, Cerna D, Scott T, Sproull M, Burgan WE, Cerra MA (2005). Enhancement of in vitro and in vivo tumor cell radiosensitivity by valproic acid. Int J Cancer.

[57] Berendsen S, Broekman M, Seute T, Snijders T, van Es C, de Vos F (2012). Valproic acid for the treatment of malignant gliomas: review of the preclinical rationale and published clinical results. Expert Opin Investig Drugs.

[58] Masoudi A, Elopre M, Amini E, Nagel ME, Ater JL, Gopalakrishnan V (2008). Influence of valproic acid on outcome of high-grade gliomas in children. Anticancer Res.

[59] Chen MY, Liao WS, Lu Z, Bornmann WG, Hennessey V, Washington MN (2011). Decitabine and suberoylanilide hydroxamic acid (SAHA) inhibit growth of ovarian cancer cell lines and xenografts while inducing expression of imprinted tumor suppressor genes, apoptosis, G2/M arrest, and autophagy. Cancer.

[60] Brodska B, Holoubek A, Otevrelova P, Kuzelova K (2013). Combined treatment with low concentrations of decitabine and SAHA causes cell death in leukemic cell lines but not in normal peripheral blood lymphocytes. Biomed Res Int.

[61] Thiemann M, Oertel S, Ehemann V, Weichert W, Stenzinger A, Bischof M (2012). In vivo efficacy of the histone deacetylase inhibitor suberoylanilide hydroxamic acid in combination with radiotherapy in a malignant rhabdoid tumor mouse model. Radiat Oncol.

[62] Blattmann C, Oertel S, Thiemann M, Weber KJ, Schmezer P, Zelezny O (2012). Suberoylanilide hydroxamic acid affects gammaH2AX expression in osteosarcoma, atypical teratoid rhabdoid tumor and normal tissue cell lines after irradiation. Strahlenther Onkol.

[63] Shay JW, Bacchetti S (1997). A survey of telomerase activity in human cancer. Eur J Cancer.

[64] Nakamura M, Masutomi K, Kyo S, Hashimoto M, Maida Y, Kanaya T (2005). Efficient inhibition of human telomerase reverse transcriptase expression by RNA interference sensitizes cancer cells to ionizing radiation and chemotherapy. Hum Gene Ther.

[65] Wong KK, Chang S, Weiler SR, Ganesan S, Chaudhuri J, Zhu C (2000). Telomere dysfunction impairs DNA repair and enhances sensitivity to ionizing radiation. Nat Genet.

[66] Akiyama M, Yamada O, Kanda N, Akita S, Kawano T, Ohno T (2002). Telomerase overexpression in K562 leukemia cells protects against apoptosis by serum deprivation and double-stranded DNA break inducing agents, but not against DNA synthesis inhibitors. Cancer Lett.

[67] Di C, Liao S, Adamson DC, Parrett TJ, Broderick DK, Shi Q (2005). Identification of OTX2 as a medulloblastoma oncogene whose product can be targeted by all-trans retinoic acid. Cancer Res.

[68] Gumireddy K, Sutton LN, Phillips PC, Reddy CD (2003). All-trans-retinoic acid-induced apoptosis in human medulloblastoma: activation of caspase-3/poly(ADP-ribose) polymerase 1 pathway. Clin Cancer Res.

[69] Fu YS, Wang Q, Ma JX, Yang XH, Wu ML, Zhang KL (2012). CRABP-II methylation: a critical determinant of retinoic acid resistance of medulloblastoma cells. Mol Oncol.

[70] Scott E, Steward WP, Gescher AJ, Brown K (2012). Resveratrol in human cancer chemoprevention--choosing the ‘right’ dose. Mol Nutr Food Res.

[71] Whitlock NC, Baek SJ (2012). The anticancer effects of resveratrol: modulation of transcription factors. Nutr Cancer.

[72] Simsek G, Tokgoz SA, Vuralkan E, Caliskan M, Besalti O, Akin I (2012). Protective effects of resveratrol on cisplatin-dependent inner-ear damage in rats. Eur Arch Otorhinolaryngol.

[73] Yu LJ, Wu ML, Li H, Chen XY, Wang Q, Sun Y (2008). Inhibition of STAT3 expression and signaling in resveratrol-differentiated medulloblastoma cells. Neoplasia.

[74] Wang Q, Li H, Wang XW, Wu DC, Chen XY, Liu J (2003). Resveratrol promotes differentiation and induces Fas-independent apoptosis of human medulloblastoma cells. Neurosci Lett.

[75] Zhang P, Li H, Wu ML, Chen XY, Kong QY, Wang XW (2006). c-Myc downregulation: a critical molecular event in resveratrol-induced cell cycle arrest and apoptosis of human medulloblastoma cells. J Neurooncol.

[76] Subbiah U, Raghunathan M (2008). Chemoprotective action of resveratrol and genistein from apoptosis induced in human peripheral blood lymphocytes. J Biomol Struct Dyn.

[77] Yamamori T, DeRicco J, Naqvi A, Hoffman TA, Mattagajasingh I, Kasuno K (2010). SIRT1 deacetylates APE1 and regulates cellular base excision repair. Nucleic Acids Res.

[78] Park HR, Kong KH, Yu BP, Mattson MP, Lee J (2012). Resveratrol inhibits the proliferation of neural progenitor cells and hippocampal neurogenesis. J Biol Chem.

[79] Badiali M, Iolascon A, Loda M, Scheithauer BW, Basso G, Trentini GP (1993). p53 gene mutations in medulloblastoma. Immunohistochemistry, gel shift analysis, and sequencing. Diagn Mol Pathol.

[80] Wang W, Kumar P, Wang W, Whalley J, Schwarz M, Malone G (1998). The mutation status of PAX3 and p53 genes in medulloblastoma. Anticancer Res.

[81] Adesina AM, Nalbantoglu J, Cavenee WK (1994). p53 gene mutation and mdm2 gene amplification are uncommon in medulloblastoma. Cancer Res.

[82] Saylors RL, Sidransky D, Friedman HS, Bigner SH, Bigner DD, Vogelstein B (1991). Infrequent p53 gene mutations in medulloblastomas. Cancer Res.

[83] Tabori U, Baskin B, Shago M, Alon N, Taylor MD, Ray PN (2010). Universal poor survival in children with medulloblastoma harboring somatic TP53 mutations. J Clin Oncol.

[84] Denissova NG, Nasello CM, Yeung PL, Tischfield JA, Brenneman MA (2012). Resveratrol protects mouse embryonic stem cells from ionizing radiation by accelerating recovery from DNA strand breakage. Carcinogenesis.

[85] Robertson K, Liner J, Meeker RB (2012). Antiretroviral neurotoxicity. J Neurovirol.

